# Maternal Mental Health During Pregnancy: A Critical Review

**DOI:** 10.7759/cureus.30656

**Published:** 2022-10-25

**Authors:** Ankit Chauhan, Jyotsna Potdar

**Affiliations:** 1 Department of Obstetrics and Gynecology, Jawaharlal Nehru Medical College, Datta Meghe Institute of Medical Sciences, Wardha, IND

**Keywords:** anxiety, postpartum depression, women, pregnancy, mental health

## Abstract

Many females have psychological well-being issues during pregnancy or after giving birth. It can happen to anybody. Depression and anxiety are the most widely recognized psychological well-being issues during pregnancy. These affect a significant number of pregnant females. Especially helpless are those females with histories of mental ailment who stop psychotropic drugs during pregnancy. Mental health issues can also be associated with abortions, broken homes, problems between the couple, unhealthy work-life balance, undue stress, physiological disorders, and other associated comorbidities. Pregnancy is traditionally considered a good time with good feelings, but it is not so. Until a few years ago, the only importance given to mental health was after childbirth (preference was given to disorders such as postpartum depression {PPD}). Nowadays, mental health is given its due attention right from conception, to antenatal care, to labor, to the postpartum period. Patients are educated on the importance of mental health and its short-term and long-term effects on the mother and the baby. The baby's father is educated because he can play a crucial role. Various researches show that children born to mothers who suffer from mental health ailments such as depression are low-birth-weight babies. Societal problems such as poverty, overpopulation, overcrowding, and poor hygiene can also adversely affect the mother's mental health. Some valuable solutions can be medication such as antidepressants, talking to a therapist, exercising, talking to close friends and family, couple counseling, and de-stressing. A phenomenon called postpartum depression is also prevalent and is given its due importance.

## Introduction and background

This review article aims to examine, analyze, and explore the factors that affect females' mental health during pregnancy, especially those deemed to negatively impact mental health. Maternal mental health during pregnancy has been broadly explored and connected with adverse results for impacted females' children. The impacts of poor mental health include preterm labor, low birth weight and early neonatal developmental disorders, adolescent neurodevelopmental issues, and young adult mental and social problems [1].

Past the possible effect on her kids, a female's mental health during pregnancy is likewise firmly associated with other parameters of her health in general. A lack of social support has a direct impact on the likelihood of experiencing despair, anxiety, and self-harm during a term. Politicians and those chipping away at the care of mothers ought to contemplate the improvement of designated social help schemes with the end goal of diminishing mental health issues among pregnant females. Pregnancy is a critical occasion for females who are in the age group of reproduction. It is enhanced by changes in hormone levels and can present itself as a period of expanded peril in the event of psychological health issues such as anxiety, depression, and self-hurt. Offering great social help to the pregnant female diminishes this risk and forestalls complications and ill effects during and after birth. In any event, no thorough study or meta-analysis has looked at the connection between prenatal mental disorders (depression, anxiety, and self-harm) and support. In light of this, it is anticipated that this systematic and analytic study will examine the connection between social support and pregnant females' mental health.

Overwhelming pressures, worries, and doubts about pregnancy, labor, the welfare of the newborn child, and the couple's future parental and nurturing responsibilities are characteristics of antenatal anxiety. Depression is the most predominant mental well-being issue during pregnancy, associated with side effects such as a discouraged state of mind, low confidence, loss of interest, sensations of uselessness, peevish temperament, loss of hunger, feelings of weakness, and lousy concentration abilities [2]. An overarching survey looking at the prevalence of prenatal depression globally revealed a range of 15%-65% and a pooled majority of 17% in poor and rich separately based on 10 distinguished systematic evaluations. One of the complicated causes of maternal deaths is self-harm during pregnancy, especially in females who have had mental health problems in the past. For instance, in a Bangladesh study, sad females who were pregnant thought about self-harm nearly 14% of the time. In high-income countries, 3%-33% of pregnant females have suicidal thoughts. According to a global survey, the prevalence of suicidal thoughts during pregnancy and after delivery ranged from 5% to 14%. Antenatal depression and anxiety badly influence a few obstetric and fetal results and, if not treated accordingly, can prompt complications, postpartum mental well-being issues, and the hazard of the debilitating relationship between the mother and the newborn child. Mental health issues during pregnancy are likewise connected with evil and unhealthy behaviors, for example, smoking and the utilization of different substances such as drugs that can, in this manner, bring about low quality of life for the mother and the child [3].

## Review

**Figure 1:**
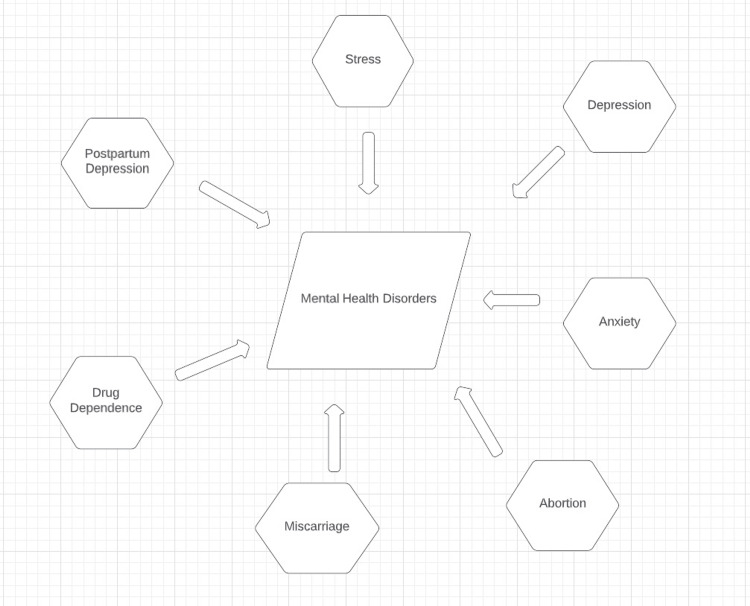
Risk factors related to maternal postpartum depression [5]

One standard methodology to help forestall or diminish the complications of pregnancy and adverse birth results as an outcome of psychological sickness is to offer solid social help to the pregnant mother. Social support is portrayed by how much social connections fill exact requirements (e.g., emotional, instrumental, tender, and additionally substantial social help) or the degree of societal harmony. The mother's positive presence in life is anticipated to grow with social support, helping to lower her risk of depression, stress, and anxiety, as well as the likelihood of a difficult pregnancy and birth. Social support can also provide a new, more effective method for pregnant females to handle difficult situations that cause stress. Numerous epidemiological studies have revealed that, during pregnancy, a lack of social support is profoundly linked to feelings of despair, anxiety, and self-harm. Be that as it may, no systematic survey and additional meta-investigation have examined and fundamentally reviewed findings from individual examinations, making the accessible proof more available to leaders and giving a gauge of the relationship between social help and mental well-being issues such as depression, anxiety, or potentially self-hurt among pregnant females. This systematic survey and meta-examination examined whether low social help is related to an expanded risk of mental well-being issues during pregnancy. Common social help is fundamentally connected with depression, anxiety, and self-hurt during pregnancy. Studies that explored the relationship between antenatal depression and social support found a positive correlation [4]. Risk factors related to maternal postpartum depression (PPD) are shown in Figure 1.

There is also a risk that abortion and miscarriage can later lead to mental disorders. The most common of these are depression, substance abuse, and anxiety. The sensational region of writing zeroing in on the utilization of prescriptions frequently has neglected to give proper respect to the possible effect of the medications. While considering the human and laboratory information on psychological instability and stress during pregnancy and the post-pregnancy time frame, it is evident that some level of exposure to medication (be it treatment or disease) consistently happens. The essential objective of the risk-benefit appraisal for treating mental illness during these periods is to help patients and their families pick the way of potential exposure that has minimal risk for them. When this choice is made, the objective is to restrict the number of exposures for the fetus [6].

Interestingly, the two sorts of insecure females fostered a weaker bonding to their fetuses and revealed a moderately high number of issues. Notwithstanding these similitudes, avoidant and anxious-ambivalent females contrasted in the methods for dealing with challenging times and in the vacillations of pre-birth attachment and psychological well-being through the various phases of pregnancy. While avoidant females would, in general, manage pregnancy-related issues by means of separation coping, anxious-ambivalent females would, in general, depend on emotion-focused coping [7]. Avoidant females showed inverted U patterns of pre-birth connection and psychological well-being, with a more grounded bond and better psychological well-being in the second trimester than in the first and third trimesters of pregnancy. Albeit anxious-ambivalent females revealed poor psychological well-being during the whole pregnancy, their bonding to the fetus expanded as the pregnancy advanced, and they eventually showed a comparatively solid bond to that reported by secure females. Albeit somewhat surprising, the changes in pre-birth connection and psychological wellness could be made sense of with regard to the general working models of insecurely attached people because avoidant females' constant method can make sense of their feeble pre-birth bonding [8]. There is broad proof that these females will generally see relationships and figures as potential wellsprings of stress and manage them by deactivating the connection and the affirmation of pain-related prompts. In particular, they will often take distance from relationships, deny needs, and smother any comprehension or feeling that can be a source of stress [9]. Factors affecting quality of life during pregnancy are shown in Table 1.

**Table 1:** Factors affecting quality of life during pregnancy [10]

Physical quality of life	Mental quality of life
Physical operative capacity	Vitality
Role: physical	Role: emotional
Bodily pain	Social support
General health	Mental health

Postpartum depression influences 13% of females around the world. The incidence is significantly higher in low- and middle-income nations, where it influences 20% of females. Its presence has been connected to aggravations in the relationship shared by the mother and the child and debilitations of comprehension, conduct, and social improvement in the kid. It slows down females' satisfaction, as well as their social participation and efficiency in their occupation [11].

Additionally, unfortunate results of this unrecognized depression are seen for the whole family, such as debilitated psychological well-being. Imminent examinations showed that 36% of females with raised adverse side effects in the early postpartum period would keep on showing negative side effects for a year and a half after labor; as far as some might be concerned, adverse side effects can persevere for as long as two years after delivery. In any case, postpartum depression after the first 12 months of life has drawn less attention than perinatal depression (i.e., during pregnancy and as long as a year postpartum) [12].

Some biological, social, demographical, and mental risk factors are known to be related to it. Past mental sickness and antenatal depression are some of the most significant. Be that as it may, the quality of the relationship with the partner has likewise been recommended as a factor for improving the depression of the mother [13].

To investigate the fundamental elements related to PPD, three perspectives, relationship fulfilment, the term of depressive episodes, and sexual fulfilment, ought to be considered. Past investigations assessing the relationship between marital disappointment and PPD have, to a great extent, been led during the first weeks, 15-18 weeks, or some months after birth [14]. Assessing relationship disappointment half a year after birth is suggested because a relationship between two partners might disintegrate following delivery because of the tremendous switches that happen during this period, particularly for parents who are having a baby for the first time. Baby care can overburden some couples during the initial stretches of life, prompting higher nurturing frustrations and stress and lower marriage fulfilment [15]. The females' impression of a decent relationship with their significant other and no revealed decrease in sex life after labor when assessed somewhere in the range of 6-8 months after delivery was related to diminished chances of persevering depressive episodes one year after delivery. A potential clarification dwells on how a female in a decent relationship with satisfaction might feel better equipped to confront the difficulties of the postpartum period [16].

The mental and actual pressure and requests related to the care of the newborn child might adversely affect moms who can depend on a decent quality of companionship with their opposite. Low-fulfilment companionships would most likely not cushion females from the factors that can prompt depression. We expect females who describe proper companionship and no intimacy challenges to have better correspondence, affection, and backing from their significant other than females who report inferior-quality companionship and intimacy issues [17]. In this manner, the outcomes concur with the marriage disagreement theory of depression, recommending that marriage problems increase the risk of psychological maladjustment. In any case, this relationship between low fulfilment and depression in mothers past the postpartum time is most likely two fanged [18]. Conjugal fulfilment is connected with social help. Females encountering such bad tendencies are bound to see their companionships adversely, experience struggle in their companionships, and use less-friendly help than females not undergoing depression. Different examinations have reported that moms who suffer from depression go through more challenges with their significant others and social relationships. In an example, depressed moms noted that they got inferior-quality help socially than revealed by non-depressed moms [19].

A subjective report also revealed the relationship between poor conjugal connections and an absence of down-to-earth help and necessary reassurance among depressed moms. Many animal models for postpartum depression apply exogenous corticosterone or models based on stress, which are consistent with the clinical findings associating stress with the risk of postpartum depression [20]. Exogenous corticosterone can promote sad and anxious-like behaviors and lead to impairments in mother care in rat models when given during pregnancy or breastfeeding. Pregnant females who experience chronic stress develop postpartum depression and anxiety-like behaviors, which reduce their ability to provide care for their babies [21]. Rehashed maternal separation has been utilized to demonstrate rodent postpartum depression and is also associated with postpartum depression. It is thought to imitate the impaired mother-infant relationship. Last but not least, stress during the initial years of life has been observed to promote depressive-like behaviors during the postpartum time period and lead to shortfalls in maternal care, which is compatible with the notion that earlier unpleasant life-altering events are a risk factor for post-partum depression [22]. With an emphasis on postpartum depression difficulties, these tests show the potential to transmit clinically important risk factors in animal models. The identification of postpartum depressive females who are at risk can be helped by a few biomarkers [23]. These are neuroendocrine (estradiol and progesterone), neurotransmitters (gamma-aminobutyric acid {GABA}, glutamate, serotonin, and dopamine), and neuroinflammatory (proinflammatory and anti-inflammatory) [24].

Studies applying functional magnetic resonance imaging (fMRI) in humans have found that postpartum depressed females' resting functional connectivity is different from that of healthy controls [25]. Even though we still don't fully understand the ramifications of these changes, it is clear that females with postpartum depression exhibit network-level changes in their brain activity. Thus, a few examples of the pathophysiological mechanisms at action are genetic, neuroinflammatory, metabolic, and network-level changes [26]. These systems interact with one another and support one another. This demonstrates that stress and past traumatic events are significant risk factors for the emergence of postpartum depression. These, including epigenetic modifications and changes in metabolic components, cause the neuroendocrine changes that are observed in it [27].

Environmental risk factors include depression, anxiety, previous adverse life events, social roles, and other associated psychiatric disorders [28]. The central umbrella risk factor for all these is stress, which is indeed found as the Everyday Stressor Index is three times higher in females with postpartum depression than in healthy controls [29]. Females who went through childhood or adulthood sexual abuse are more susceptible to developing postpartum depression, as much as three times more than females who did not experience childhood or adulthood sexual abuse [30].

The physician's role in health supervision

It is, therefore, pertinent to broaden the mother-child dyadic gaze and also consider the father in perinatal health checkups, thus grasping that a contextual and relational reality, rather than being in isolation, is what makes mental health what it is [31]. The recognition of the significance of including parents as relevant figures in the health and development of their children is crucial in this setting, and pediatricians and family doctors who treat children play a key role in doing so [32].

Good ways to involve the father in the care of health are showing encouragement for going to the health check [33], talking to the couple, showing how offspring view their parents as figures to look up to [34], and educating parents on how to support the mother in breastfeeding, emphasizing the distinct role that each of them plays in upbringing [35]. Since the first year of a baby's existence is when the roots of his future mental health are set [36], the systemic view becomes crucial when diagnosing and intervening [37], fundamentally in the early stages of the kid. Regardless of the type of relationship that exists between the parental couple, it is essential to provide timely diagnosis and treatment for both paternal and mother postpartum depression [38,39] in order to encourage the practice of responsible parenting and family well-being [40].

## Conclusions

This review article has comprehensively covered all the tangible factors that can directly or indirectly affect females mental health during pregnancy, from antenatal, perinatal, and postnatal causes to abortion, miscarriage, and the quality of the relationship of the mother and the father. Associated sequelae such as drug dependence, stress, depression, and anxiety have also been covered. The ideal management of these mental health disorders has been given due importance. Postpartum depression is a significant disorder suffered by females after giving birth in the postpartum period. Due diligence has been shown to commonly used medications during pregnancy and antidepressants in the postpartum period so that the incidence of drug abuse and drug dependence are minimized and the adverse effects are tolerated better. Educating the mother and the father about mental health issues during and after pregnancy is also critical as some prospective parents aren't aware of them. It is imperative as it can affect the mother and the baby, leading to developmental disorders, low birth weights, polyhydramnios, oligohydramnios, etc. Social support, mother-child bonding, and medications have been discussed as treatment remedies along with common diagnostic criteria. Positive relationships were found among various risk factors and predictors of poor mental health during and after pregnancy, and the associated treatment modalities such as therapy and medications were discussed accordingly. Further areas of research pertaining to this topic could be the effects of modern lifestyle on maternal mental health, for example, smart phones. There is a lack of relevant data in this regard, and this could be the topic of interest for future researches.

## References

[1] Ahmad M, Vismara L (2021). The psychological impact of COVID-19 pandemic on women’s mental health during pregnancy: a rapid evidence review. Int J Environ Res Public Health.

[2] López-Morales H, Del Valle MV, Canet-Juric L, Andrés ML, Galli JI, Poó F, Urquijo S (2021). Mental health of pregnant women during the COVID-19 pandemic: a longitudinal study. Psychiatry Res.

[3] Bedaso A, Adams J, Peng W, Sibbritt D (2021). The relationship between social support and mental health problems during pregnancy: a systematic review and meta-analysis. Reprod Health.

[4] Divney AA, Sipsma H, Gordon D, Niccolai L, Magriples U, Kershaw T (2012). Depression during pregnancy among young couples: the effect of personal and partner experiences of stressors and the buffering effects of social relationships. J Pediatr Adolesc Gynecol.

[5] Davalos DB, Yadon CA, Tregellas HC (2012). Untreated prenatal maternal depression and the potential risks to offspring: a review. Arch Womens Ment Health.

[6] Diego MA, Jones NA, Field T, Hernandez-Reif M, Schanberg S, Kuhn C, Gonzalez-Garcia A (2006). Maternal psychological distress, prenatal cortisol, and fetal weight. Psychosom Med.

[7] Brown SJ, Yelland JS, Sutherland GA, Baghurst PA, Robinson JS (2011). Stressful life events, social health issues and low birthweight in an Australian population-based birth cohort: challenges and opportunities in antenatal care. BMC Public Health.

[8] Dunkel Schetter C (2011). Psychological science on pregnancy: stress processes, biopsychosocial models, and emerging research issues. Annu Rev Psychol.

[9] Rich-Edwards JW, Kleinman K, Abrams A, Harlow BL, McLaughlin TJ, Joffe H, Gillman MW (2006). Sociodemographic predictors of antenatal and postpartum depressive symptoms among women in a medical group practice. J Epidemiol Community Health.

[10] Martin CR, Jomeen J (2010). Assessment of quality of life during pregnancy and in the postnatal period. Handbook of disease burdens and quality of life measures.

[11] Cheng D, Schwarz EB, Douglas E, Horon I (2009). Unintended pregnancy and associated maternal preconception, prenatal and postpartum behaviors. Contraception.

[12] Gipson JD, Koenig MA, Hindin MJ (2008). The effects of unintended pregnancy on infant, child, and parental health: a review of the literature. Stud Fam Plann.

[13] Washington CI, Jamshidi R, Thung SF, Nayeri UA, Caughey AB, Werner EF (2015). Timing of postpartum intrauterine device placement: a cost-effectiveness analysis. Fertil Steril.

[14] Pawluski J, Dickens M (2019). Pregnancy: a final frontier in mental health research. Arch Womens Ment Health.

[15] Barba-Müller E, Craddock S, Carmona S, Hoekzema E (2019). Brain plasticity in pregnancy and the postpartum period: links to maternal caregiving and mental health. Arch Womens Ment Health.

[16] Payne JL, Maguire J (2019). Pathophysiological mechanisms implicated in postpartum depression. Front Neuroendocrinol.

[17] Lindahl V, Pearson JL, Colpe L (2005). Prevalence of suicidality during pregnancy and the postpartum. Arch Womens Ment Health.

[18] Feldman R, Granat A, Pariente C, Kanety H, Kuint J, Gilboa-Schechtman E (2009). Maternal depression and anxiety across the postpartum year and infant social engagement, fear regulation, and stress reactivity. J Am Acad Child Adolesc Psychiatry.

[19] Halligan SL, Murray L, Martins C, Cooper PJ (2007). Maternal depression and psychiatric outcomes in adolescent offspring: a 13-year longitudinal study. J Affect Disord.

[20] Lyons-Ruth K, Zoll D, Connell D, Grunebaum HU (1986). The depressed mother and her one-year-old infant: environment, interaction, attachment, and infant development. New Dir Child Dev.

[21] Murray L (1992). The impact of postnatal depression on infant development. J Child Psychol Psychiatry.

[22] Murray L, Cooper PJ (1997). Postpartum depression and child development. Psychol Med.

[23] Righetti-Veltema M, Conne-Perréard E, Bousquet A, Manzano J (2002). Postpartum depression and mother-infant relationship at 3 months old. J Affect Disord.

[24] Skalkidou A, Hellgren C, Comasco E, Sylvén S, Sundström Poromaa I (2012). Biological aspects of postpartum depression. Womens Health (Lond).

[25] Wisner KL, Stowe ZN (1997). Psychobiology of postpartum mood disorders. Semin Reprod Endocrinol.

[26] Say L, Chou D, Gemmill A (2014). Global causes of maternal death: a WHO systematic analysis. Lancet Glob Health.

[27] Huizink AC, Mulder EJ, Robles de Medina PG, Visser GH, Buitelaar JK (2004). Is pregnancy anxiety a distinctive syndrome?. Early Hum Dev.

[28] Hernández-Martínez C, Val VA, Murphy M, Busquets PC, Sans JC (2011). Relation between positive and negative maternal emotional states and obstetrical outcomes. Women Health.

[29] Nieminen K, Stephansson O, Ryding EL (2009). Women's fear of childbirth and preference for cesarean section--a cross-sectional study at various stages of pregnancy in Sweden. Acta Obstet Gynecol Scand.

[30] Teixeira C, Figueiredo B, Conde A, Pacheco A, Costa R (2009). Anxiety and depression during pregnancy in women and men. J Affect Disord.

[31] Dennis CL, Falah-Hassani K, Shiri R (2017). Prevalence of antenatal and postnatal anxiety: systematic review and meta-analysis. Br J Psychiatry.

[32] (1996). The global burden of disease: a comprehensive assessment of mortality and disability from diseases, injuries, and risk factors in 1990 and projected to 2020: summary. https://apps.who.int/iris/bitstream/handle/10665/41864/0965546608_eng.pdf.

[33] Dadi AF, Miller ER, Bisetegn TA, Mwanri L (2020). Global burden of antenatal depression and its association with adverse birth outcomes: an umbrella review. BMC Public Health.

[34] Gausia K, Fisher C, Ali M, Oosthuizen J (2009). Antenatal depression and suicidal ideation among rural Bangladeshi women: a community-based study. Arch Womens Ment Health.

[35] Frautschi S, Cerulli A, Maine D (1994). Suicide during pregnancy and its neglect as a component of maternal mortality. Int J Gynecol Obstet.

[36] Satyanarayana VA, Lukose A, Srinivasan K (2011). Maternal mental health in pregnancy and child behavior. Indian J Psychiatry.

[37] Fisher J, deMello MC, Izutsu T (2009). Mental health aspects of pregnancy, childbirth and the postpartum period. Contemporary topics in women's mental health-global perspectives.

[38] Chandra PS (2009). The interface between reproductive health and psychiatry. Contemporary topics in women's mental health-global perspectives.

[39] van Bussel JC, Spitz B, Demyttenaere K (2006). Women's mental health before, during, and after pregnancy: a population-based controlled cohort study. Birth.

[40] Gavin NI, Gaynes BN, Lohr KN, Meltzer-Brody S, Gartlehner G, Swinson T (2005). Perinatal depression: a systematic review of prevalence and incidence. Obstet Gynecol.

